# The Multifaceted Role of Th1, Th9, and Th17 Cells in Immune Checkpoint Inhibition Therapy

**DOI:** 10.3389/fimmu.2021.625667

**Published:** 2021-03-12

**Authors:** Jongdae Lee, Beatriz Lozano-Ruiz, Fengyuan Mandy Yang, Dengxia Denise Fan, Liya Shen, Jose M. González-Navajas

**Affiliations:** ^1^ School of Basic Medical Sciences and the State Key Laboratory of Respiratory Disease, Guangzhou Medical University, Guangzhou, China; ^2^ Alicante Institute for Health and Biomedical Research (ISABIAL), Hospital General Universitario de Alicante, Alicante, Spain; ^3^ Networked Biomedical Research Center for Hepatic and Digestive Diseases (CIBERehd), Institute of Health Carlos III, Madrid, Spain; ^4^ Department of Pharmacology, Pediatrics and Organic Chemistry, University Miguel Hernández, Elche, Spain; ^5^ Institute of Research, Development and Innovation in Healthcare Biotechnology in Elche (IDiBE), University Miguel Hernández, Elche, Spain

**Keywords:** T helper (Th) cell, immune checkpoint inhibition, CTLA-4, PD-1, cancer therapy, Th1, Th17, Th9

## Abstract

During the last decade, immune checkpoint inhibition (ICI) has become a pillar of cancer therapy. Antibodies targeting CTLA-4 or PD-1/PD-L1 have been approved in several malignancies, with thousands of clinical trials currently underway. While the majority of cancer immunotherapies have traditionally focused on enhancing cytotoxic responses by CD8^+^ or NK cells, there are clear evidences that CD4^+^ T cell responses can modulate the immune response against tumors and influence the efficacy of ICI therapy. CD4^+^ T cells can differentiate into several subsets of helper T cells (Th) or regulatory T cells (Treg), with a wide range of effector and/or regulatory functions. Importantly, different Th subsets may have different and sometimes contrasting roles in the clinical response to ICI therapy, which in addition may vary depending on the organ and tumor niche. In this review, we discuss recent evidence that highlights how ICI therapy impacts Th1, Th9, and Th17 cells and *vice versa*. These data might be important designing better interventions that unleash the full potential of immune response against cancer.

## Introduction

The effector mechanisms of both innate and adaptive immunity are important in the response against tumors. While much of the research on the adaptive immune response to tumors has focused on CD8^+^ cytotoxic T lymphocytes (CTL), the importance of CD4^+^ T helper (Th) cells has been traditionally underappreciated. However, CD4^+^ Th cells constitute an essential part of the immune system due to their ability to interact with and regulate other immune cells through cell-to-cell contacts and cytokine secretion. Effector Th cells may participate in the antitumor immune response in various ways, either directly by eliminating tumor cells or indirectly by providing cytokines and co-stimulatory signals that improve the efficacy of CTL responses (1). In contrast, a different subset of CD4^+^ T cells, called regulatory T (Treg) cells, are known for their ability to restrain effector T cell responses and may also suppress the immune response to tumors (reviewed in another article of this issue) (2).

The majority of current anticancer therapies rely on drugs that either kill dividing cells or prevent cell division, which usually come at a high price and cause adverse effects on healthy cells and tissues. The host immunity to tumors, on the other hand, may be highly specific for tumor antigens and not injure normal cells or tissues. Therefore, immunotherapy has the potential of being the most tumor-specific treatment that can be conceived. The earliest attempts to harness the immune system to fight tumors trace back to the late 19th century with the works of Wilhelm Busch, Friedrich Fehleisen and William B. Coley among others. Busch (3) and Fehleisen (4) realized that some cancer patients experienced notable tumor regression after developing erysipelas, a skin infection usually caused by *Streptococcus pyogenes*. Busch was actually the first to inoculate bacteria as a therapy for cancer in 1868 (5). Coley continued and expanded this work during the 1890s. He reported new cases of tumor regression upon contraction of erysipelas (6) and began treating bone and soft tissue sarcomas first with live streptococcal cultures and later, after two infection-related deaths, with heat-killed *Streptococcus* and *Serratia* mixtures (the famous Coley’s toxins) (7). Although it was criticized at the time because of inconsistent results, this initial form of immunotherapy was used for the next 40 years until it was replaced by chemotherapy and radiotherapy treatments. It was not until the end of the 20th century and beginning of the 21st that the field of immunotherapy was revitalized with several key discoveries, including the identification of tumor-associated antigens, the use of recombinant cytokines such as IL-2, tumor-specific monoclonal antibodies, adoptive cell therapy with tumor-infiltrating lymphocytes (TILs) (8), dendritic cell vaccines (9), chimeric antigen receptor (CAR) T cells (10), and immune checkpoint inhibitors (ICI) (11). Among these, ICI therapy and CAR T cell therapy are perhaps the most promising, as they have achieved surprising results in subsets of patients with several malignancies that had limited therapeutic options. However, ICI therapy is beneficial only to a small fraction of cancer patients (12, 13), therefore there is still much to understand to unleash the full potential of ICI therapy.

In this review, we will discuss recent evidences that highlight the importance of Th cells in the efficacy of ICI therapy. Th1, Th2, Th9, Th17 and T follicular helper (T_FH_) cells have been broadly studied in cancer immunotherapy. However, due to length limitations, we will focus on Th1, Th9 and Th17 cells.

## ICI Therapy: A Paradigm Shift in Cancer Treatment

The notion that certain molecules expressed on the surface of T cells may function as immune brakes dates back to the 1990s. Cytotoxic T cell antigen 4 (CTLA-4) was first described in 1987 by Brunet et al. (14), but its role as a negative regulator of T cell proliferation and function was not demonstrated until 1995 by the group of J.P. Allison (15) and the generation of CTLA-4–deficient mice (16). CTLA-4 is a receptor that structurally belongs to the immunoglobulin superfamily and is homologous to CD28 (14, 17), the main co-stimulatory receptor on T cells. It is mainly expressed on CD4^+^ and CD8^+^ T cells upon activation, but is constitutively expressed on Tregs where it contributes to their suppressive function (18). Like CD28, CTLA-4 binds to CD80 (B7-1) and CD86 (B7-2) molecules on APCs, but with much higher avidity than CD28 (17) and with opposite effect (15, 19).

Programmed cell death 1 (PD-1) was identified in 1992 by the group of T. Honjo (20), who also proved its role as a negative regulator of immune responses (21, 22). PD-1 is expressed on activated T and B cells and other myeloid cells, and is bound by PD-ligand 1 (PD-L1) and PD-L2, which also belong to the B7 family. PD-L1 (also known as B7 homolog 1, or B7-H1) is constitutively expressed on myeloid cells and is inducible in many other cell types, including tumor cells, in the presence of inflammatory signals (23). Compared to PD-L1, basal expression of PD-L2 (also known as B7-DC) is low and mainly restricted to dendritic cells and activated macrophages, although its expression can be induced in other immune cells and non-immune cells under certain stimuli (24). Akin to PD-L1, PD-L2 may be also expressed by tumor cells (25). In addition to CTLA-4 and PD-1, other inhibitory molecules have been also discovered, such as TIM3 (T cell immunoglobulin and mucin-domain containing 3) (26), LAG-3 (lymphocyte activation gene 3) (27, 28), or TIGIT (T cell immunoglobulin and ITIM domain) (29, 30).

These inhibitory molecules are generally referred to as immune checkpoints and are crucial to maintaining self-tolerance, preventing autoimmunity, and controlling the duration and extent of immune responses in order to minimize collateral tissue damage. The antibodies that block them are therefore known as immune checkpoint inhibitors (ICIs). ICI therapy was born based on the hypothesis that blocking the negative signals provided by CTLA-4 and PD-1 with monoclonal antibodies (mAb) could unleash the T cell-dependent immune response against cancer. The remarkable results of these therapies in animal models and clinical trials led to the first approval, in 2011, of an anti–CTLA-4 mAb (Ipilimumab) for the treatment of advanced melanoma. This was followed by the approval of other mAbs targeting PD-1 (Nivolumab and Pembrolizumab) and PD-L1 (Atezolizumab, Durvalumab) in 2014 and 2016 respectively. These therapies have changed the pattern of treatment and the outcome for certain groups of patients with advanced cancers. The U.S. food and drug administration (FDA) has already approved the use of these therapies in at least 15 cancer types (31), including advanced melanoma, lung cancer, renal cell carcinoma, urothelial cancer, liver cancer or squamous cell carcinoma. In addition, there are currently hundreds of clinical trials, including more than 30 registered phase III studies, testing the efficacy of ICIs in different types of cancer (www.clinicaltrials.gov). A clear example of this paradigm shift is the fact that, at present, nearly all patients diagnosed with metastatic lung cancer receive PD-L1 blockade as part of their treatment (32).

However, ICI therapy still presents two main drawbacks: the high percentage of patients that do not respond to therapy, and the development of immune-related adverse events due to an overactive immune response. Intensive experimental research is now focused on deciphering the mechanisms of action with the aim of overcoming these drawbacks. In addition, clinical trials are testing anti–CTLA-4, anti–PD-1, or anti–PD-L1 mAbs in combination with each other and with other agents, trying to find therapeutic regimes with improved efficacy and safety.

## Brief Overview of Th Cell Development and Differentiation

**Figure 1:**
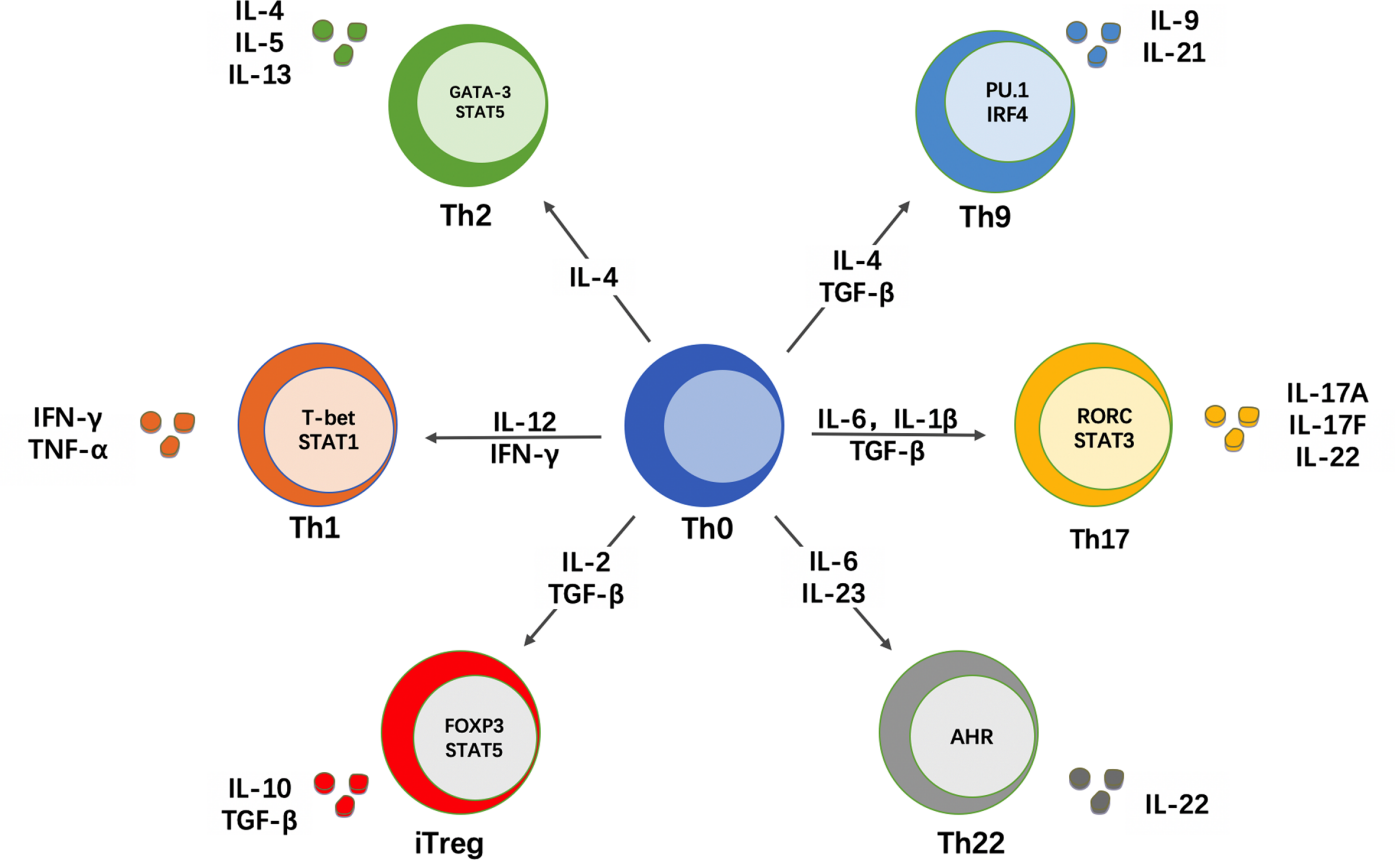
CD4^+^ T cell differentiation in different helper subsets. Naïve CD4^+^ T cells (Th0) can differentiate into different subsets of T helper (Th) cells. This differentiation depends on the cytokine milieu that is present during antigen recognition and TCR stimulation. In response to these signals, naïve CD4^+^ T cells upregulate key transcription factors that control subset differentiation, which in turn determines the production of signature cytokines that mediate the effector function of each Th subset.

CD4^+^ T cells express a T cell receptor (TCR) that recognizes peptide antigens presented by class II major histocompatibility complex (MHC-II) molecules on the surface of antigen-presenting cells (APCs). The CD4 molecule is a co-receptor that binds to the beta-chain of the MHC-II molecule and facilitates the interaction of the TCR with the peptide-MHC-II complex (33). T lymphocytes originate from pluripotent precursor cells that arise in the fetal liver or adult bone marrow and mature in the thymus, where they are referred to as thymocytes. CD4^+^ T cells that successfully undergo this thymic maturation process become naïve T cells (Th0). Therefore, naïve CD4^+^ T cells are cells that, by definition, have not yet encountered antigen and lack effector functions. Naïve T cells are activated by a “two-signal” interaction with APCs. First, their TCR recognizes peptide antigens presented by MHC-II molecules on APCs and, second, they receive co-stimulatory signals from other molecules on the surface of these APCs. Upon activation, naïve T cells have the ability to proliferate and differentiate into one of several subsets of effector Th cells or Treg cells. Subset differentiation depends on the cytokine milieu that is present during TCR activation, which drives the upregulation of key transcription factors that in turn regulate the expression of specific surface markers and effector cytokines associated with each subset (34, 35). In addition to the cytokine environment, the strength of the TCR signal has been also shown to regulate the differentiation program of naïve CD4^+^ T cells (36). At least 6 subsets of effector Th cells exist, namely Th1, Th2, Th9, Th17, Th22 and T follicular helper (T_FH_) cells (**Figure 1**). It was initially thought that the differentiation of naïve T cells into different lineages was an irreversible event. However, ample evidence now supports that these subsets, particularly Th17 and Treg cells, are not terminally differentiated cells and retain a certain degree of plasticity, allowing for conversion to other lineages under particular stimulation or pathogenic conditions (37, 38). Another classical concept in immunology was that naïve T cells are a homogeneous population of uncommitted precursor cells that differ only in their TCR specificity (34). However, this concept has been also challenged by data showing that some naïve T cells are already pre-committed to a specific subset, with Th17 and Treg cells again on the spotlight (39, 40).

## The Multifaceted Roles of Th Subsets in ICI Therapy

There is still much to learn about the immunological mechanisms and impact of ICI therapy in many tumors. It is nonetheless becoming clear that successful immunotherapy-induced anti-tumor immune responses requires both CD8^+^ and CD4^+^ T cells. A recent report by Alspach et al. showed that this is true even in tumors that do not express MHC-II molecules (41). The authors also found that ICI therapy was more effective when tumor cells express both MHC-I and MHC-II neoantigens, i.e., they are targets for both CD8^+^ and CD4^+^ cells (41). CD4^+^ cells support multiple CTL functions including clonal expansion, cytotoxicity, and their ability to infiltrate tumors [reviewed by Borst et al. (1)]. At the molecular level, CD4^+^ T cell help instills a transcriptomic signature in CTLs that not only promotes their cytotoxic or migratory potential, but also downregulates the expression of inhibitory receptors such as PD-1 or LAG-3 (42). Mechanistically, it was shown that this help was largely mediated by the interaction of CD4^+^ cells with DCs, causing upregulation of CD70 on the DCs that in turn relay the costimulatory signal through CD70-CD27 interactions on the CTLs (42). In addition, the impact of CD4^+^ T cells on the transcriptional profile of memory CTLs has been recently identified (43). Using a vaccination strategy that excludes or includes “help” signals and genome-wide analyses, the authors show that CD4^+^ T cell help during CTL priming supports the expansion of the T central memory (T_CM_) and T effector memory (T_EM_) subpopulations, as well as the upregulation of many genes associated with cytotoxic function (including granzyme B, perforin or IFN-γ) within the T_EM_ population. Remarkably, the authors also showed that memory CTLs generated in the presence of help signals acquire epigenetic traits that allow them to respond more vigorously upon subsequent antigen-independent cytokine stimulation or antigen-dependent but help-independent re-challenge (43).

These and other studies (1) reflect the importance of CD4^+^ T cell responses in the tumor microenvironment (TME). However, different Th subsets may have distinct or even contrasting roles in the clinical response to ICI therapy (**Table 1**). In addition, the expression pattern of some immune checkpoints may vary among different Th subsets. A recent report showed that Th1 and Th2 cells display a higher expression of CTLA-4 compared to Th17 cells in healthy subjects and in patients with multiple sclerosis or lupus (71). Based on heatmap representation of flow cytometry data, the expression of LAG-3 seemed also higher in Th2 cells compared to Th1 or Th17 cells, at least in healthy subjects, whereas the expression of other immune checkpoints including PD-1 or TIGIT remained mostly unchanged in these three subject groups (71). However, the differential expression of immune checkpoints in different Th subsets from cancer patients is an interesting question that remains unexplored. Last, it is important to identify the differences that may exist within different organs and tumor niches. In the following sections, we discuss the contribution of Th1, Th9, and Th17 subsets to the success of ICI therapy.

**Table 1:** Summary of positive and negative effects of Th1, Th9, and Th17 subsets in the efficacy of ICI therapy.

CD4^+^ Th subset	Human or mouse study	Effect on ICI therapy	References
**Th1**			
	Mouse	Prolonged IFN-γ signaling mediates both PD-L1-dependent and -independent resistance to ICI therapy	(44)
	Human	IFN-γ-related gene expression signatures predict responses to PD-1 and CTLA-4 blockade in melanoma and other tumors	(45, 46)
	Mouse	Combination of CTLA-4 and PDL-1 blockade increases IFN-γ production and enhances tumor rejection in melanoma	(47)
	Human and mouse	Deficiency in IFN-γ signaling impairs melanoma tumor rejection after CTLA-4 blockade	(48)
	Human and mouse	Blockade or genetic deletion of CTLA-4, alone or in combination with PD-1 blockade, expands a population of ICOS^+^ Th1-like cells	(49, 50)
	Mouse	Activation of ICOS signaling and high frequency of ICOS^+^ Th1-like cells correlates with higher tumor rejection after CTLA-4 blockade	(51, 52)
	Mouse	IFN-γ impairs Treg function, activates CD103^+^ DCs that present tumor antigens, and induces polarization of iNOS^+^ macrophages	(53–55)
	Human	High frequency of Th1 cells is associated with IRAEs in the skin and the gastrointestinal tract of patients treated with ICI inhibitors	(56–58)
**Th17**			
	Mouse	Th17 polarization reduces the efficacy of CTLA-4 and PD-1 combination blockade in bone metastases.	(59)
	Mouse	Concomitant blockade of TGFβ signaling and CTLA-4 or PD-1 reduces Th17 differentiation and promotes melanoma and breast cancer tumor rejection	(60)
	Human	Patients with stage IV melanoma that respond to anti–PD-1 therapy present with higher frequencies of IL-17A^+^CD4^+^ cells	(61)
	Mouse	HDAC6-deficient Th17 cells enhance the production of IFN-γ by CD8^+^ T cells. Deletion of HDAC6 in CD4^+^ T cells promotes HCC rejection upon PD-1 blockade	(62)
	Mouse	IL-21 derived from Th17 cells increases the frequency of intratumoral CX3CR1^+^CD8^+^ T cells, which improves melanoma tumor rejection	(63)
	Human	Th17 cells are associated with IRAEs in patients with melanoma receiving anti–CTLA-4 therapy	(64, 65)
**Th9**			
	Human and mouse	Th9 cells are increased in melanoma patients that respond to PD-1 therapy. IL-9 favors the cytotoxic function of mouse CD8^+^ T cells	(66)
	Human	Infiltration of IL-9^+^ cells in the TME correlated with exhausted phenotype of CD8^+^ cells, but it favored the response to anti–PD-1 therapy in bladder cancer	(67)
	Human and mouse	Th9 cells infiltrate CRC in humans and are associated with higher CD8^+^ cell frequency. PD-1 blockade enhances IL-9 production in human CRC and mouse HCC.	(68, 69)
	Mouse	IL-21 derived from Th9 cells induces the production of IFN-γ by CD8^+^ cells	(70)

CRC, colorectal carcinoma; CTLA-4, cytotoxic T cell antigen 4; DCs, dendritic cells; HCC, hepatocellular carcinoma; HDAC6, histone deacetylase 6; ICI, immune checkpoint inhibition; ICOS, inducible co-stimulator; IFN, interferon; iNOS, inducible nitric oxide synthase; IRAEs, immune-related adverse events; PD-1, programmed death 1; PD-L1, programmed death ligand 1; TGF, tumor growth factor; TME, tumor microenvironment.

### Th1 Cells and ICI Therapy

Th1 cells are generated when naïve T cells are activated in the presence of IL-12 and express the T-box transcription factor TBX21 (T-bet), which induces the prototypical Th1 cytokine IFN-γ (72). In addition to Th1 cells, IFN-γ can be secreted by CD8^+^ T cells, γδ T cells, NK cells and, to a lesser extent, by NKT cells and APCs (73). IFN-γ is one of the most intensively studied cytokines in the field of cancer biology. It plays a major role in anticancer immunity by promoting the activity of CTL and NK cells, as well as by upregulating MHC expression and antigen presentation by dendritic cells. Moreover, IFN-γ inhibits Treg function and promotes the differentiation of macrophages towards a more pro-inflammatory and tumoricidal M1 phenotype (73). However, IFN-γ may also have pro-tumorigenic effects. One important negative effect is that cancer cells may express PD-L1 after exposure to IFN-γ, impairing antitumor immunity (74). In a sense, this evasion mechanism developed by tumors emphasizes the importance of this cytokine during T cell immunosurveillance. Accordingly, IFN-γ production in the TME could have negative effects with immunotherapy treatments that do not block PD-1/PD-L1 pathway. Conversely, when therapeutic regimens incorporate anti–PD-1/PD-L1 agents, the presence of IFN-γ could correlate with better response and overall survival (75–77). Nevertheless, positive and negative effects of this Th1-associated cytokine in ICI therapy have been reported, and recent reports show that the influence of IFN-γ in cancer immunotherapy goes beyond the PD-1/PD-L1 pathway.

A study by Benci et al. showed that prolonged IFN signaling may promote both PD-L1-dependent and -independent resistance to ICI therapy and to combination of ICI with radiation (44). Importantly, both IFN-γ (type II) and type I IFN signaling seemed to have a similar effect, causing tumors to enhance the expression of ligands for multiple T cell inhibitory receptors such as TIM3 and LAG-3 (44). These results support the notion that persistent exposure to IFN contributes not only to PD-L1 expression in tumor cells but also to PD-L1-independent evasion mechanisms. On the other hand, a study by Ayers et al. identified that a TME characterized by active IFN-γ signaling is a common feature of tumors that respond to PD-1 blockade with Pembrolizumab (45). The authors demonstrate that a set of IFN-γ-responsive genes associated with antigen presentation, cytotoxic T cell responses and chemokine expression were necessary for clinical benefit in up to 9 different cancer types (45). Similarly, upregulation of an IFN-γ-responsive gene expression signature appears to be important for the clinical success of anti–CTLA-4 mAb, as evidenced by the analysis of tumor specimens from melanoma patients treated with Ipilimumab (46). Consistently, combination therapy of anti–CTLA-4 and anti–PDL-1 mAbs plus an anticancer vaccine resulted in a significant increase in IFN-γ production by both CD4^+^ and CD8^+^ cells, which correlated with higher rates of melanoma tumor rejection (47). In fact, a surprisingly high proportion (9 out of 12) of metastatic melanoma patients that do not respond to Ipilimumab harbor tumors with loss of IFN-γ signaling (48). Furthermore, mice bearing melanoma tumors deficient in IFN-γ receptor 1 (IFNGR1) had impaired tumor rejection after CTLA-4 blockade (48).

**Figure 2:**
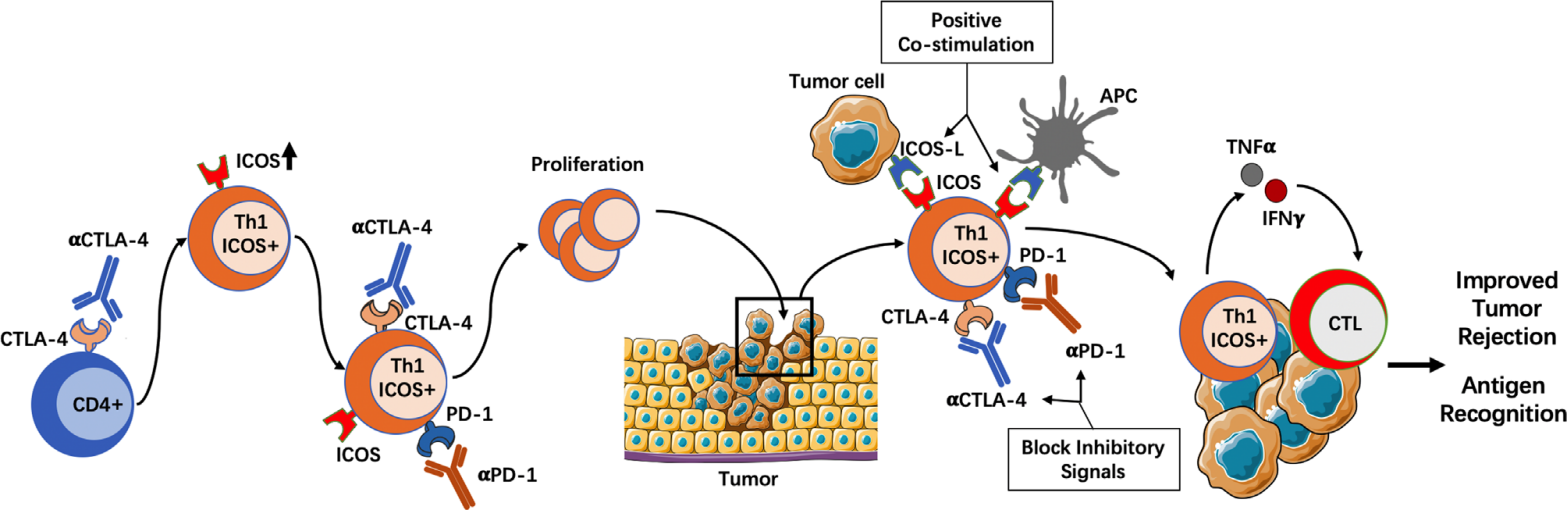
The importance of ICOS^+^ Th1 cells in ICI therapy. Genetic absence of CTLA-4 or blocking with anti–CTLA-4 antibody induces the differentiation of ICOS^+^ Th1-like cells. Additionally, concomitant PD-1 blockade may promote the proliferation of these ICOS^+^ T cells, which have the ability to migrate to tumors. In the tumor microenvironment, ICOS pathway activation by APCs or tumor cells induces positive co-stimulation, while CTLA-4 and/or PD-1 blockade prevents negative co-stimulatory signals. The result is a population of tumor-infiltrating CD4^+^ T cells with high expression of IFN-γ and TNF-α, which can recognize tumor antigens and boost the antitumor immune response. APC, Antigen presenting cell; CTLA-4, Cytotoxic T cell antigen 4; ICOS, Inducible co-stimulator; IFN, Interferon; PD-1, Programmed death 1; TNF, Tumor necrosis factor.

The expression of inducible co-stimulator (ICOS) seems to be an important marker of Th1-associated antitumor response. This was first identified in a clinical trial for the use of Ipilimumab in bladder cancer patients (49). Although this trial did not allow correlation with clinical outcome, it did show that CD4^+^ T cells from blood and tumor tissue of all treated patients increased the expression of ICOS and the production of IFN-γ. These CD4^+^ICOS^hi^IFN-γ-producing cells were able to recognize tumor antigens and their expansion increased the ratio of Teff/Treg cells in peripheral blood and tumors (49). Subsequent studies further demonstrated the antitumor role of ICOS^+^ Th1-like effector T cells. In mice bearing melanoma tumors, ICOS^+^ cells comprised a population of tumor-specific and Th1 cytokine-producing effector cells (78). Moreover, in ICOS- or ICOS ligand (ICOSL)-deficient mice, the efficacy of anti–CTLA-4 therapy was significantly diminished, although the specific contribution of ICOS^+^CD4^+^ cells versus ICOS^+^CD8^+^ cells in tumor rejection was not addressed in this study (78). Additionally, concomitant CTLA-4 blockade and ICOS engagement by tumor cells that express ICOSL significantly improved rejection of established melanoma and prostate cancer in mice (51). Notably, this therapeutic combination gave rise to a population of tumor-infiltrating CD4^+^ T cells with high expression of IFN-γ and TNF-α in response to re-stimulation with tumor antigens, suggesting that these cells were potent tumor antigen-specific Th1 cells (51). Moreover, IFN-γ was indispensable for tumor protection since IFN-γ-receptor deficient mice lost the survival benefit of this therapeutic combination (51). The importance of ICOS^+^ Th1 cells was further highlighted in another study by Wei et al. (50). Using mass cytometry and computational approaches, the authors show that CTLA-4 not only attenuates T cell activation but also regulates CD4^+^ T cell differentiation in mice, but not CD8^+^ differentiation. Both the genetic absence of CTLA-4 and anti–CTLA-4 therapy led to the development of non-canonical ICOS^+^ Th1-like effector cells (50). When comparing anti–CTLA-4 and anti–PD-1 monotherapies, it seems that anti–CTLA-4 treatment is more effective expanding the ICOS^+^CD4^+^ Th1 compartment than anti–PD-1 treatment (79). Interestingly, this Th1 population also upregulates PD-1, which may limit further expansion (52). In fact, when anti–CTLA-4 and anti–PD-1 are administered together there is a further increase in the frequency of these Th1-like effector cells compared with anti–CTLA-4 monotherapy (52). As in prior observations, a higher frequency of these Th1-like cells correlated with higher tumor rejection in a colon adenocarcinoma xenograft model (52). Altogether, these data suggest that ICOS^+^CD4^+^ Th1-like effector cells play an important role in the response to ICI therapy (**Figure 2**).

In addition to IFN-γ, the Th1-related cytokine IL-12 is also important for the immune response in general, and for ICI therapy in particular. Using intravital real-time imaging and single-cell RNA sequencing analysis, Garris et al. elegantly showed that effective anti–PD-1 therapy in mice requires IL-12 production by intratumoral DCs (80). Similarly, intratumoral delivery of IL-12 in combination with IL-7, by a tumor-selective oncolytic vaccinia virus, culminated in tumor rejection and increased multiple antitumor immune pathways (81). Moreover, this strategy further increased the anti-tumor efficacy of PD-1 or CTLA-4 blockade in previously resistant tumors (81). Additional mechanisms whereby Th1 cells can improve ICI therapy include the control of Treg cells, which contribute to tumor tolerance, or the activation of specific subsets of myeloid cells. A study by Overacre-Delgoffe et al. showed that IFN-γ can induce Treg “fragility,” defined as loss of function *ex vivo* and loss of tumor tolerance *in vivo*, and that IFN-γ-induced Treg fragility is required for effective anti–PD-1 therapy in a colon adenocarcinoma model (53). It has been also shown that combination of PD-1 and CTLA-4 blockade directly triggers a Th1-like response that activates the tumor-infiltrating CD103^+^ DCs (54), which are highly efficient at presenting tumor antigens and show enhanced IL-12 production. In a positive feedback loop, IL-12 production by these cells boosted T-bet expression and IFN-γ production by intra-tumoral CD4^+^ T cells which increased the therapeutic effect of anti–CTLA-4/anti–PD-1 therapy (54). Recent findings provided further evidence of the modulation of specific myeloid subsets by Th1-associated cytokines during anti–PD-1/anti–CTLA-4 combination therapy. Using single-cell RNAseq and cytometry by time of flight (CyTOF), Gubin et al. observed multiple subpopulations of monocytes/macrophages that changed overtime in a manner partially dependent of IFN-γ (55). Of note, the authors show that IFN-γ production in the TME by revitalized CD4^+^ cells drives polarization of newly arrived monocytes into iNOS^+^ pro-inflammatory macrophages, which contribute to tumor rejection (55). Together, these results support the hypothesis that modulation of tumor-infiltrating myeloid cells by IFN-γ-producing Th1 effector cells is at least partially responsible for the success of ICI combination therapy.

### Th17 Cells and ICI Therapy

Th17 cells play divergent roles in carcinogenesis, and whether they promote or inhibit cancer seem to be dependent on the type of the tumor or the anatomical localization. The pro-tumorigenic role of Th17 cells in carcinogenesis is often related to chronic inflammation. It is widely accepted that excessive inflammation from Th17 cells may play important pathogenic functions in several inflammation-associated cancers, including colon, lung and liver cancers (82–85). On the other hand, antitumor functions of Th17 cells have also been reported, mainly through the recruitment and activation of neutrophils, NK cells and CTLs into the TME (86).

With respect to ICI therapy, the role of Th17 cells is not fully uncovered and both positive and negative effects have been reported. As discussed above, ICI therapy seems to expand the Th1 population in different tumor types. However, tissue-specific conditions or differences in the tumor niche may skew the differentiation of CD4^+^ T cells towards other Th subsets. A recent paper by Jiao et al. showed that, in patients with metastatic prostate cancer, Ipilimumab enhanced the Th1 subset signature in soft tissue metastases, but expanded the Th17 lineage in bone metastases (59). In addition, the authors injected prostate tumor cells into mice either subcutaneously or intraosseously, as models of soft tissue metastases or bone metastases respectively, and evaluated the effect of anti–CTLA-4 plus anti–PD-1 combination therapy in both models. When tumor cells were injected subcutaneously, the treatment resulted in an increase of intra-tumoral Th1 populations and improved survival. When tumor cells were injected into the bone, intra-tumoral T cells were polarized to Th17 rather than Th1, and the treatment had minimal effects on tumor volume or survival (59). Mechanistically, the authors showed that bone tumors promote excessive bone resorption, which results in high amounts of TGF-β within the TME. In turn, TGF-β may restrain Th1 differentiation and, in the presence of IL-6, promote Th17 polarization. Consistent with this hypothesis, concomitant blockade of TGF-β potentiated the efficacy of ICI therapy by restoring Th1 lineage polarization in the bone tumors (59). Very similar conclusions can be taken from another study in which the importance of TGF-β, both in the efficacy of ICI therapy and in the differentiation of Th1 and Th17 cells within the TME, was demonstrated by the use of bifunctional antibody-ligand traps (60). These traps are comprised of an anti–CTLA-4 or anti–PD-L1 antibody fused to a TGF-β receptor II ectodomain, so they simultaneously block one of the immune checkpoints and TGF-β signaling in the target cell. Anti–CTLA-4/TGF-βRII trap was able to abrogate the differentiation of CD4^+^ T cells into Th17 cells and switch them to IFN-γ-producing Th1 cells (60). In addition, both anti–CTLA-4/TGF-βRII and anti–PD-L1/TGF-βRII traps were more effective in inhibiting tumor progression compared with CTLA-4 or PD-L1 monotherapy in melanoma and breast cancer mouse models, which was associated with an elevation in tumor-reactive IFN-γ-expressing CD8^+^ cells and a reduction in Treg cells (60). Together, both studies (59, 60) suggest that driving CD4^+^ T cells away from Treg or Th17 phenotype to a Th1 phenotype may improve the efficacy of ICI therapy by enabling effective activation of antitumor CD8^+^ T cells. This concept is also supported by data from colorectal cancer (CRC) studies, both in humans and mouse models. In mice, IL-17 signals directly within transformed colonic epithelial cells to promote their proliferation and early tumor development (87) and to inhibit their production of CXC chemokine ligand 9 (CXCL9) and CXCL10 (88). These chemokines are recognized by the CXCR3 receptor, which mediates the migration of CD8^+^ T cells to sites of inflammation and tumors (89, 90). Therefore, by blocking CXCL9/10 production, IL-17 inhibits the infiltration of CD8^+^ CTLs into CRC and reduces antitumor immunity (88). A similar observation has been recently reported in humans (91). IL-17A levels were increased in sera from patients with advance-stage CRC, which was associated with downregulated CXCR3 expression on CD8^+^ T cells. Furthermore, the presence of Th17 cells in the TME was negatively associated with the presence of CXCR3^+^CD8^+^ cells. Noteworthy, patients with high levels of IL-17A and low CXCR3 expression on CD8^+^ T cells had worse prognosis (91).

However, the notion that the Th17/IL-17 axis is always associated with poor responses in cancer immunotherapy has been challenged by other studies. Krieg et al. (61) characterized the immune cell subsets in the peripheral blood of patients with stage IV melanoma receiving anti–PD-1 therapy and analyzed the differences between responders and non-responders. After therapy, T cells from responders presented with higher frequencies for IL-17A along with other markers of T cell activation such as IFN-γ, PD-1 and granzyme B (61). Anker et al. (92) used an uropathogenic strain of *Escherichia coli*, known as CP1, to study whether the immunostimulatory properties of bacteria can be utilized to enhance immunotherapies in immunologically “cold” tumors such as prostate cancer. Use of CP1 in combination with PD-1 blockade increased survival and decreased tumor burden, whereas anti–PD-1 monotherapy did not (92). This therapeutic effect coincided with tumor infiltration by multiple anti-tumor immune cell types including IFN-γ-producing CD8^+^ cells, M1-polarized macrophages, and NK cells. Within the CD4^+^ T cell compartment, CP1 increased infiltration of Th17 cells with a corresponding decrease of Treg cells (92), although the specific contribution of Th17 cells to the therapeutic effect was not addressed. Qiu et al. (62) showed that a subset of histone deacetylase 6 (HDAC6)-deficient Th17 cells enhanced the production of IFN-γ by CD8^+^ T cells, which correlated with higher PD-1 and PD-L1 expression in hepatocellular carcinoma (HCC) tumor cells. Consequently, specific deletion of HDAC6 in total CD4^+^ T cells resulted in stronger antitumor response and HCC tumor rejection upon anti–PD-1 treatment in mice, although this effect was only partially attributable to IL-17 (62).

Beyond IL-17, other cytokines that can be produced by Th17 cells, such as IL-21 or IL-22, may also be important in ICI therapy. In muscle-invasive bladder cancer, the presence of IL-22^+^ cells in the tumor was associated with poor prognosis and increased expression of exhaustion markers such as PD-1, CTLA-4, TIM3, and LAG-3 in CD8^+^ T cells, but also with a better response to Nivolumab in *in vitro* assays with freshly resected tumor tissue (93). However, whether IL-22 expression could be used as a predictor for the response to Nivolumab in these patients still needs to be investigated in prospective clinical studies. Recently, using single-cell RNA sequencing and various models of mixed bone marrow chimera and T cell adoptive transfer in mice, Zander et al. demonstrated that IL-21 derived from CD4^+^ T cells has a critical role in promoting the formation of CX3CR1^+^ CD8^+^ T cells, a subset of CD8^+^ T cells with potent cytolytic activity during viral infections (63). Moreover, using the B16-F10 melanoma model, the authors showed that IL-21 produced by *in vitro* differentiated Th17 cells also increases the frequency of CX3CR1^+^ CD8^+^ T cells in the tumor, which in turn was associated with reduced tumor burden (63). IL-21 is also secreted by T_FH_ and Th9 cells, and therefore it will be discussed in more detail later.

Altogether, the role of Th17 cells in cancer immunotherapy is complex, as it is in carcinogenesis as well. Whether Th17 cells have a positive or negative influence on ICI therapy may be dependent on the tumor type, the severity of the disease, or differences within the TME. Another important factor that may contribute to such functional diversity is their plasticity. Th17 cells are probably the most plastic of the Th subsets. For example, Th17 cells can acquire Th1-like characteristics and the ability to secrete high amounts of IFN-γ (37, 94), which would likely play a role in enhancing antitumor immune responses. On the other hand, Th17 cells can also transdifferentiate into suppressive IL-17^+^Foxp3^+^ or IL-17^−^Foxp3^+^ Treg cells (95), serving as a source of tumor-associated Treg cells. Such plasticity complicates the therapeutic use of these cells, but it may serve as a valuable strategy to enhance cancer immunotherapies.

### Th9 Cells and ICI Therapy

IL-9-producing Th cells (Th9) comprise a relatively new T cell subset that, despite being able to co-produce large quantities of IL-10, has been implicated in tissue inflammation and immunity against parasites (96, 97). The presence of Th9 cells in the TME of solid tumors is associated with a robust anti-tumor immune response through both innate and adaptive immune mechanisms, which have been reviewed recently (98). With regards to ICI therapy, a study with 46 melanoma patients treated with Nivolumab (anti–PD-1 Ab) showed a significant increase in the frequency of Th9 cells in the peripheral blood of those patients that responded to the therapy, while other Th subsets remained unchanged between responders and non-responders (66). Blocking IL-9 signaling reduced the expression of granzyme B and perforin in human CD8^+^ T cells, and stimulation with recombinant IL-9 enhanced the cytotoxicity of tumor-specific mouse CD8^+^ T cells (66). In addition, blocking IL-9 *in vivo* promoted tumor progression in the B16 melanoma xenograft model and in the Braf/Pten model, in which tumors are developed *de novo* in the mouse skin (66). Paradoxically, in muscle-invasive bladder cancer patients, higher infiltration of IL-9^+^ cells in the tumor tissue correlated with impaired cytotoxic function of CD8^+^ T cells and NK cells, higher frequencies of Treg cells, and poor prognosis (67). These CD8^+^ T cells showed an exhausted phenotype with high expression of PD-1, TIM3, and LAG-3. However, after PD-1 blockade with Nivolumab, these cells regained their proliferative and cytotoxic potential *in vitro*, measured by higher expression of granzyme B and perforin, while CD8^+^ T cells from tumor samples with low infiltration of IL-9^+^ cells did not respond to anti–PD-1 treatment (67). These *in vitro* results suggest that the presence of Th9 cells could be a predictive marker for the use of anti–PD-1 therapy in bladder cancer patients, although this hypothesis should be tested in clinical or *in vivo* studies. Anti–PD-1 therapy can also have a direct effect on Th9 cells. A recent study using tumor samples from CRC patients detected the presence of PD-1^+^ Th9 cells within the TIL population, which showed a positive correlation with the frequency of CD8^+^ cells (68). Moreover, PD-1 engagement suppressed IL-9 production by these cells, which was restored after PD-1 blockade (68). In mice, higher plasma levels of IL-9 were also found after PD-1 blockade in an orthotopic model of HCC (69). Therefore, it might be speculated that ICI therapy increases the anti-tumor response of Th9 cells in CRC or HCC.

In the presence of IL-1β, Th9 cells secrete large quantities of IL-21 (70), a pleiotropic cytokine that also possesses anticancer properties. For example, IL-21 derived from Th9 cells induces the production of IFN-γ by CD8^+^ and NK cells, which was required for the anti-tumor effects of Th9 cells (70). A recent study evaluated the importance of intra-tumoral delivery of IL-21 using tumor cell lines expressing human EGFR (hEGFR) epitope and anti-hEGFR monoclonal antibody (Erbitux)-IL-21 fusion protein (Erb-IL-21) (99). Erb-IL-21 administration increased the frequency of IFN-γ^+^CD8^+^ T cells in the TME and reduced the overall expression of PD-1 in these cells. This reduction in PD-1 expression was attributed to the expansion of PD-1^int^TIM3^-^CD8^+^ cells and a reduction in the proliferation of PD-1^+^TIM3^+^CD8^+^ population (99). Congruently, intra-tumoral IL-21 administration enhanced the efficacy of both anti–PD-1 and anti–CTLA-4 therapy in mice bearing colon adenocarcinoma xenograft tumors (99). However, it is important to note that IL-21 can be produced by other T cell populations, including Th17 cells (as discussed above) and T_FH_ cells, and therefore its effects may not always be attributable to a particular Th subset. Identifying the source of IL-21 would provide additional information on the immune profile of the TME.

In summary, Th9 cells may play an important role in the response to ICI therapy. These data provide the rationale to investigate the use of adoptive transfer of Th9 cells in combination with ICI therapy, or as a predictive biomarker for the efficacy of ICI treatments.

## Th Cells as Important Mediators of Immune-Related Adverse Events (IRAEs)

Cancer patients receiving ICI therapy may suffer from a diverse array of immune-related adverse events (IRAEs), specially at the skin, gastrointestinal tract, liver and endocrine system. These reactions may vary from mild to severe or even fatal (100), and they can precipitate the abandonment of the therapy even after favorable responses have been observed. Both the success and the adverse events of ICI therapy are the result of an invigorated immune system, and therefore over-activated CD4^+^ Th subsets may be also involved in such IRAEs. The epidemiology and pathophysiology of IRAEs has been the subject of several recent reviews (100, 101), and therefore we will focus only on recent data that reflects the role of Th subsets on these negative immune reactions.

A higher number of Th1 cells, compared to Th2 and Treg cells, was present in skin lesions of cancer patients who developed lichenoid dermatitis after treatment with Nivolumab, Pembrolizumab or a combination of Ipilimumab and Nivolumab (56). These results suggest that Th1 cells may mediate the appearance of inflammatory skin lesions, although other Th subsets such as Th9 or Th17 cells were not analyzed in this study. Moreover, Th1 cells seem mostly associated with gastrointestinal adverse events. Analysis of tissue samples from melanoma patients with Ipilimumab-associated colitis revealed IFNγ as the highest expressed inflammatory cytokine in the colonic mucosa of these patients (57). Similarly, severe colitis with robust infiltration of T-bet^+^ Th1 cells was recently documented in patients with metastatic melanoma after Nivolumab treatment (58). TNF-α, which can be produced by activated Th1 cells, is also well known for its involvement in IRAEs. Indeed, anti-TNF-α agents such as infliximab (anti-TNF-α antibody) are commonly used, after initial corticosteroid therapy, for the management of ICI-related colitis. This has been supported by several studies showing that blocking TNF-α ameliorates gastrointestinal IRAEs without affecting the efficacy of ICI therapy. Using different mouse models of intestinal inflammation exacerbated by combined anti–PD-1 and anti–CTLA-4 therapy, Pérez-Ruiz et al. showed that prophylactic or concurrent blockade of TNF-α ameliorates the development of colitis without affecting the antitumor response (102). In addition, blocking TNF-α with infliximab, after tapering of glucocorticoid therapy, also reduced colitis in cancer patients treated with different ICI inhibitors without affecting their efficacy (103). These results were corroborated in a large cohort of melanoma patients receiving infliximab for the management of severe ICI-related colitis (104).

Th17 cells have been also associated with adverse events of the ICI therapy. It was reported that Th17 cells were increased after CTLA-4 blockade in patients with metastatic melanoma, although there were no differences between responder and non-responder patients indicating that Th17 cells did not participate in the response to therapy (64). Importantly, this increase in Th17 cells was driven mostly by patients with IRAEs, suggesting a possible role for Th17 cells in ICI-induced toxicities. Similar results were obtained in another study performed in melanoma patients treated with ipilimumab, in which pre-treatment IL-17 levels were associated with the development of severe intestinal inflammation (65).

In summary, Th1 and/or Th17 cells may be implicated in the inflammatory adverse events that are associated with ICI therapy. The role of Th9 cells in these adverse events has not been explored, but based on the reported effects of ICI therapy on the Th9 population and the potent anti-tumor activity triggered by these cells, it could be speculated that over-activation of Th9 cells may also contribute to the development of IRAEs.

## Concluding Remarks

For many years, immunotherapy approaches have focused on studying the direct antitumor effect of cytotoxic cells, such as CTLs and NK cells, while the role of CD4^+^ T cells have remained somewhat underappreciated. However, the recent literature discussed herein shows how different subsets of Th cells may respond differently to immune checkpoint blockade and how they affect the efficacy of this therapy. It seems therefore clear that successful ICI therapy requires appropriate effector Th responses. Among all Th subsets, Th1 or Th1-like cells seem to be the most beneficial. In particular, ICOS^+^ Th1-like effector cells, which arise after CTLA-4 or CTLA-4 plus PD-1 blockade, are important for the therapeutic effect of Ipilimumab and are associated with longer survival. Therefore, therapeutic approaches to activate the ICOS pathway in combination with immune checkpoint blockade represents a promising option. CD4^+^ T cell plasticity is another important element that should be further investigated in the context of ICI therapy. For many years it was believed that CD4^+^ T cells differentiate along discrete pathways that dictate their effector functions. However, we know now that the process of T cell differentiation is more fluid, and that certain Th cell subsets can change their phenotype to acquire traits of a different Th cell or Treg cells. Understanding the mechanisms of T cell plasticity will provide additional opportunities to enhance the efficacy of ICI therapy, for example by inducing the conversion of other Th subsets into Th1 cells or reducing their transformation into Treg cells.

ICI therapy is becoming a therapeutic option for a growing number of malignancies. Moreover, the use of ICIs in combination with radiation, chemotherapy or surgery is also the subject of many ongoing clinical trials in melanoma, non-small cell lung cancer and other cancers. Using the immune system to fight cancer has, at least, three major advantages: specificity, memory and adaptability. There are also obstacles, such as the appearance of adverse reactions or IRAEs. In addition, these therapies still fail to work in a significant proportion of patients. Studying the impact of other therapeutic agents on the immune system, identifying reliable predictive biomarkers, targeting new molecules to improve efficacy, and dissecting the cellular and molecular mechanisms whereby CD4^+^ T cells are involved in the antitumor effect are some critical issues that need to be resolved for further clinical development of ICI therapy.

## Author Contributions

All authors contributed significantly to the drafting and editing of this manuscript. JL and JG-N conceived the manuscript idea and revised the manuscript content. BL-R, FY, DF, and LS created the manuscript tables and figures. All authors contributed to the article and approved the submitted version.

## Funding

This work was supported by grant PI19/01554 from the Instituto Nacional de Salud Carlos III (co-financed with FEDER funds), Madrid, Spain; grant CDEI-03/20-A from Generalitat Valenciana, Valencia, Spain to JG-N; and Guangzhou Medical University Startup Fund to JL.

## Conflict of Interest

The authors declare that the research was conducted in the absence of any commercial or financial relationships that could be construed as a potential conflict of interest.
